# Psychological Resources and Biomarkers of Health in the Context of Chronic Parenting Stress

**DOI:** 10.1007/s12529-021-10007-z

**Published:** 2021-08-06

**Authors:** Alexandra D. Crosswell, Sara Sagui-Henson, Aric A. Prather, Michael Coccia, Michael R. Irwin, Elissa S. Epel

**Affiliations:** 1 Department of Psychiatry and Behavioral Sciences, University of California, San Francisco, 3333 California Street Suite 465, CA 94118 San Francisco, USA; 2 Osher Center for Integrative Medicine, University of California, San Fransisco, CA San Francisco, USA; 3 Cousins Center for Psychoneuroimmunology, Semel Institute for Neuroscience and Human Behavior, and Department of Psychiatry and Biobehavioral Sciences, University of California, Los Angeles, CA Los Angeles, USA

**Keywords:** Psychological resources, Well-being, Self-acceptance, Stress, Biomarkers, Metabolic health

## Abstract

**Background:**

Epidemiological studies link psychological resources to better physical health. One reason may be that psychological resources are protective in stressful contexts. This study tested whether indeed psychological resources are protective against biological degradation for healthy mid-life women under the chronic stress of caring for a child with an autism spectrum disorder diagnosis (“caregivers”).

**Methods:**

We tested whether five types of psychosocial resources (i.e., eudaimonic well-being, autonomy, purpose in life, self-acceptance, and mastery) were associated with biological indices of aging in a sample of mid-life women stratified by chronic stress; half were caregivers (*n* = 92) and half were mothers of neurotypical children (*n* = 91; controls). Selected stress and age related biological outcomes were insulin resistance (HOMA-IR), systemic inflammation (IL-6, CRP), and cellular aging (leukocyte telomere length). We tested whether each resource was associated with these biomarkers, and whether caregiving status and high parenting stress moderated that relationship.

**Results:**

All the psychological resources except mastery were significantly negatively associated with insulin resistance, while none were related to systemic inflammation or telomere length. The relationships between eudaimonic well-being and HOMA-IR, and self-acceptance and HOMA-IR, were moderated by parental stress; lower resources were associated with higher insulin resistance, but only for women reporting high parental stress. The well-known predictors of age and BMI accounted for 46% of variance in insulin resistance, and psychological resources accounted for an additional 13% of variance.

**Conclusion:**

These findings suggest that higher eudaimonic well-being and greater self-acceptance may be protective for the metabolic health of mid-life women, and particularly in the context of high parenting stress. This has important implications given the rising rates of both parental stress and metabolic disease, and because psychological interventions can increase eudaimonic well-being and self-acceptance.

**Supplementary Information:**

The online version contains supplementary material available at 10.1007/s12529-021-10007-z.

## Introduction

With the increasing rates of chronic illness, mental health diagnoses, and psychological stress in the USA and across the globe, increasing individuals’ psychological resources to best cope with these environmental demands is of urgent importance for public health. Psychological resources are aspects of one’s personality or appraisals of life circumstances that are thought to provide a reserve, or resource, for people to draw from and promote resilience during challenging life demands [1]. Examples of these resources include a sense of control over one’s life and purpose in life. Higher levels of these individual-level characteristics are associated with lower risk of clinical health problems [2, 3], and have shown to buffer against stress-related increases in systemic inflammation [4–6].

The construct of psychological resources is relatively broad, encompassing numerous more specific and definable categories. Unpacking the construct of psychosocial resources is important in order to identify their mechanistic relationships to clinical health outcomes. One approach to this is to look to the original model of the dimensions of psychological well-being proposed by Carol D. Ryff [7]. In this model, six measurable components were identified: the belief that one’s life has purpose, meaning, and direction (purpose in life); the ability to act from an internal set of standards versus external pressures (autonomy); the capacity to effectively manage current life circumstances (mastery); acceptance of oneself, including knowledge and acceptance of the good and bad parts of themselves (self-acceptance); positive social relationships (social support); and a sense of continued development of themselves (personal growth). There is debate regarding the precision of these six dimensions [8], and Ryff has subsequently refined the model [9, 10]. Yet, this original model maintains its utility by providing an approach with clear measurement recommendations and providing a solid foundation for conceptual thinking and discussion in the domain of positive psychological well-being. Several of the constructs we examine in this study are pulled from this model.

A related and overlapping construct that has been a focus of study in health research is eudaimonic well-being. Eudaimonic well-being (or eudaimonia) is the psychological experience of feeling a deep sense of satisfaction that comes from self-realization and pursuing a noble life purpose beyond self-gratification [11]. It refers to the evaluative judgments one makes about their own lives, such as whether they believe personal talents and abilities are being realized, how they are functioning socially, and whether they have a positive perspective on the way society at large is functioning. It is distinct from another key component of well-being—hedonic well-being (sometimes called emotional well-being)—which encompasses experiences of positive emotions, including joy and happiness [12].

Growing evidence suggests that greater eudaimonic well-being may be associated with better biological functioning, as measured with immune, cardiovascular, and metabolic biomarkers [13, 14]. Specifically, greater eudaimonic well-being has been associated with lower levels of systemic inflammation, such as interleukin-6 (IL-6) and C-reactive protein (CRP) [14–18], and lower blood glucose levels [2], though not all studies have supported this [19]. Many of the positive associations have been found in large epidemiological studies which provides strong empirical support for at least cross-sectional associations [e.g., 2, 20–23]. Furthermore, replicated studies have shown that greater eudaimonic well-being (and not hedonic well-being) is associated with a decreased expression of the stress-related gene expression profile known as the “conserved transcriptional response to adversity” (CTRA) which includes the up-regulation of pro-inflammatory genes and down-regulation of genes involved in the antibody response [24–27]. Recent evidence has also demonstrated that psychosocial interventions that increase eudaimonic well-being also decrease the CTRA gene expression profiles, lending further support to the direct impact of eudaimonia on biological functioning [28, 29].

Eudaimonia, along with other psychosocial resources, has been linked to better clinical health outcomes such as reduced risk of metabolic syndrome [2, 21], cardiovascular disease [30, 31], and premature mortality [20, 32]. One of the primary theoretical arguments for the association between psychological resources and better physical health is that greater resources provide a stress buffer that aid in reducing the negative impact that chronic and acute stressors have on emotions and behaviors [1, 10, 33]. Mastery, positive social relationships, and purpose in life, each help increase persistence through challenging tasks [34–36], and increased self-acceptance improves emotion regulation [37]. Eudiamonic well-being also appears to offer a protective buffer against negative health risks in the context of socioeconomic disadvantage [6, 10]. Identifying stress buffers that can be targeted with non-pharmaceutical treatments is particularly relevant in the USA where subjective stress levels are rising. A report from the American Psychological Association found that nearly 20% of adults say that their mental health is worse than it was 1 year ago [38]. In particular, parents of children < 18 years old living at home are suffering, reporting significantly greater increases in stress and decreases in mental health compared to non-parents or parents of older children [39]. The type of psychological resource which is particularly protective for chronically stressed parents in terms of preventing biological degradation is unknown and a focus of the current paper.

Another need in the current literature is to expand the examination of psychological resources and stress-related biomarkers of disease beyond markers of systemic inflammation. Insulin sensitivity is a compelling outcome to examine given it is a strong indicator of metabolic regulatory health, and epidemiological data has linked eudaimonic well-being with lower risk of metabolic syndrome [2]. Telomere length is another compelling biomarker to examine, given that a significant body of literature has documented associations between negative psychological states and traits with shorter telomere length [40–43], while only a handful of studies have explored associations between positive psychological traits and telomere length. The initial studies in this area have had mixed results, suggesting further inquiry is needed. For example, a positive association between social support and telomere length in one study [43] was not replicated in another with similar sample demographic profiles [44]. Several studies have found associations between positive psychological resources like optimism, dispositional mindfulness, and self-compassion with longer telomere length [45, 46], though other studies, including one that utilized data from two national cohort studies, have reported null associations [41, 47]. The literature linking well-being to telomere length is bolstered by several small positive psychological intervention trials which have reported stabilized telomere length in the intervention group, an effect researchers hypothesize is driven by an increase in overall well-being and stress resilience (for a review, see Conklin et al. [48]).

The current study examined whether specific types of psychological resources (i.e., eudaimonic well-being, autonomy, purpose in life, self-acceptance, and mastery) are associated with established biological risk factors of future disease that are relevant for younger healthy women, and whether these resources are particularly important in the context of chronic stress. We recruited women experiencing both high self-reported subjective stress and high objective stress as they are mothers of children with an autism spectrum disorder, and an age-matched group of mothers who reported low subjective stress and had a similarly aged child without a developmental delay. Caregiving for a loved one with a disability or disease has been one model that psychological scientists use to investigate experiences of chronic stress because caregivers report high subjective stress and a greater number of daily stressors [49]. Moreover, given the increase in perceived parental stress due to the quality of life changes caused by the current global pandemic [39], parenting young children has become a chronic stressor for many.

A body of literature has similarly examined parents of children with developmental or mental health disorders to compare them to parents of children without diagnosed disorders on biological and psychological outcomes. These studies have generally demonstrated that parenting a child with a disability is associated with worse mental and physical health. For example, parenting a child with a disability is associated with more daily stressors [50], greater risk of clinical depression [51], greater negative affect, and lower positive affect [52], worse self-reported health [52], decreased episodic memory [53], and higher morbidity [54], compared to parenting a child with no diagnosed disorders, though these relationships often were moderated by individual-level factors (e.g., age, gender, whether they live with child or not). Past research has also demonstrated that in addition to exposure (whether one is caregiving or not), the perception of the experience, such as levels of perceived stress, caregiving burden, and amount of external support, also matter for health. Specifically, higher levels of perceived stress in parents of children with autism have been associated with biological alterations that may be markers or mechanisms of increased disease risk including shorter immune cell telomeres [55], and fewer formal and informal support services have been associated with higher systemic inflammation [56], though not all studies have found this [57, 58].

Relevant for this study, positive psychological resources can strengthen parents’ ability to cope. Research shows that higher levels of acceptance [59], social support [60], and having an internal locus of control [59], are associated with increased mental and physical resilience in parents with non-neurotypically developing children. An important distinction between the current study design and the body of work of non-normative parenting is that this study had inclusion criteria that caregiving mothers had to report high levels of perceived stress in addition to the objective fact of having a child with a diagnosed disorder. This is because the purpose of our study was to investigate eudaimonic well-being in the context of chronic stress (using maternal caregivers as a model of this), and chronic stress is a state characterized by the subjective experience of overwhelm and negative affect reported in measures of perceived stress.

In this study, we examine how five psychological resources relate to biomarkers of aging and disease that have previously been associated with both positive well-being indices and chronic stress and have high variability within healthy mid-life women. These biomarker measures are systemic inflammation (IL-6, CRP), insulin sensitivity, and telomere length. We examined these relationships in a sample of 183 women that were recruited because of their chronic stress status; half of the women (*n* = 92) were mothers caring for a child with an autism spectrum disorder, and the other half (caregivers; *n* = 91) were women who were caring for a similarly aged child with current neurotypical development. We hypothesized that (1) across the sample as a whole, greater psychological resources would be associated with better biological functioning, specifically: lower levels of HOMA-IR, lower levels of IL-6 and CRP, and longer telomere length; (2) compared to the control group, caregivers to children with autism would have worse mental and physical health, as indexed by greater parenting stress, lower resources, higher HOMA-IR, higher IL-6 and CRP, and shorter telomere length; (3) compared to caregivers with low resources, caregivers with high resources will have lower systemic inflammation, lower HOMA-IR, and longer telomere length.

## Methods

### Participants

Participants were recruited in the San Francisco Bay Area via mass mailings, announcements in parenting publications, flyers and notices posted in local schools, and recruitment from the University of California San Francisco (UCSF) Autism Clinic, advertising particularly to mothers of children with autism to examine their psychological experience. Eligibility requirements included being between 20 and 50 years old with at least one child between the ages of 2 and 16, and because of the influence of these factors on biomarkers, a non-smoker, and free from current psychiatric illness (self-reported). For chronically stressed mothers, additional inclusion criteria were caring for a child diagnosed with autism spectrum disorder and a Perceived Stress Scale [61] score ≥ 13 to ensure participants had high subjective stress. For control participants, additional inclusion criteria were caring for a neurologically typical child and a Perceived Stress Scale score ≤ 19.

### Procedures

Participants were tracked longitudinally over 2 years (between 2011 and 2014). At baseline, then 9, 18, and 24 months later, participants completed in-person lab visits with psychological questionnaires and a blood draw. Measures of well-being were only completed at the 18-month assessment and thus the data from that visit is used here for well-being measures and for biomarkers HOMA-IR, CRP, IL-6, and telomere length. The demographic data we present is taken from the baseline assessment, as that is when demographic information was collected. The UCSF institutional review board approved this research, and informed consent was obtained from all participants.

### Psychological Resource Measures

Psychological resources were assessed using the following measures: the Mental Health Continuum Short Form Scale (MHC-SF) [62, 63], three subscales from the Psychological Well-Being Scales (Ryff’s original measure [7]; autonomy, purpose in life, and self-acceptance), and the Pearlin Mastery Scale to capture mastery [64]. All scales were standardized via Z-scores for analysis so that comparisons between the measures could be made.

#### The Mental Health Continuum Short Form Scale (MHC-SF)

The MHC-SF [65] is a 14-item scale that measures emotional well-being (3 items), psychological well-being (6 items), and social well-being (5 items). Several of the items in this scale overlap with the Psychological Well-Being Scales developed by Ryff [7] and are described in the following section. This study focuses on eudaimonic well-being in both private and community life and thus utilized the approach of previous studies by combining the psychological and social well-being subscales into a single scale of eudaimonic well-being (11 items total) [26, 65]. These items were designed to capture functioning in life versus feelings toward life as other well-being measures do, consistent with Keyes et al. [66] description of eudaimonic well-being. Example items are as follows: how often did you feel you had something to contribute to society? How often did you feel that you had warm and trusting relationships with others? How often did you feel that your life has a sense of direction or meaning to it? The response scale is from *never* (0) to *everyday* (5), and the prompt asks participants to think about how they felt over the past month. Items were summed and averaged, with higher scores indicating greater eudaimonic well-being. This scale had adequate reliability, with α of 0.84.

#### Psychological Well-Being Scales

To capture other positive mental health functioning constructs, we used 27 items selected from the Psychological Well-Being Scales (PWBS) developed by Ryff [67]; the specific items that were selected were those from the subscales of autonomy, purpose in life, and self-acceptance. The nine-item autonomy subscale captures how much a person operates from their own set of standards instead of by the standards set by others (example item: My decisions are not usually influenced by what everyone else is doing). The nine-item purpose in life subscale captures how much one has goals and a sense of meaning in life (example item: I enjoy making plans for the future and working to make them a reality). The nine-item self‐acceptance scale assesses positive attitude toward the self and acceptance of both good and bad qualities of the self (example item: When I look at the story of my life, I am pleased with how things have turned out). The response scale for these items is from *strongly disagree* (1) to *strongly agree* (6).

#### Pearlin Mastery Scale

The Pearlin Mastery Scale was used to capture mastery [64], which is the extent to which someone believes their life outcomes are under their personal control versus controlled by outside forces or a victim of fate. An example item is: I have little control over the things that happen to me. The response scale is from *don’t agree at all* (1) to *agree very much* (4). Conceptually this scale measures a variation of mastery different from that described by Ryff in her original model. Ryff’s description of environmental mastery (which was not captured in this study) captures how much an individual feels they can and have been able to create living environments that meet their needs and capacities.

### Parenting Stress

The subjective stress specific to one’s role as a parent was captured with the Parental Stress Scale [68]. This 18-item scale captures positive components (emotional benefits, self-enrichment, personal development) and negative components (demands on resources, opportunity costs, and restrictions) of the experience of being a parent. Example items are: I am happy in my role as a parent; If I had to do it again I might decide not to have children; I enjoy spending time with my children; I feel overwhelmed by the responsibility of being a parent. The response scale options are *strongly disagree* (1), *disagree* (2), *undecided* (3), *agree* (4), and *strongly agree* (5). The stem of the items asks participants to respond to the items “in terms of how your relationship with your child or children typically is.” The positive items were reverse coded, and then the items were summed to create a total score. High total scores indicate higher parental stress.

### Perceived Stress

The 10-item Perceived Stress Scale (PSS) [61] was used to measure general perceptions of stress in one’s current life over the past month, regardless of the source of stress. The response scale options are *never* (0), *almost never* (1), *sometimes* (2), *fairly often* (3), and *very often* (4). The positive items were reverse coded, and then the items were summed to create a total score with a range from 0 to 40. High total scores indicate higher global perceived stress. Cronbach’s alpha was 0.87 at baseline.

### Biological Measures

At each assessment, a registered nurse collected 200 mL of blood in EDTA tubes via venipuncture, which was then placed on ice, centrifuged for acquisition of plasma, and stored at −80 °C for subsequent batch testing.

The Homeostatic Model Assessment for Insulin Resistance (HOMA-IR) was calculated as fasting insulin (µU/ml) × glucose (mg/dL) × 0.00247 following previously set standards [69].

C-reactive protein (CRP) levels were determined by a high sensitivity ELISA (R&D Systems, Minneapolis, MN) according to the manufacturer’s protocol, with an extended standard curve to a lower limit of detection of 0.2 mg/L. Circulating levels of IL-6 were determined using a high sensitivity ELISA (R&D Systems, Minneapolis, MN), with a lower limit of detection of 0.2 pg/mL. All samples were run in duplicate. The intra- and inter-assay precision of all tests was < 8%. Because of skewed distributions, we normalized CRP and IL-6; natural log values were taken after adding constant +1 to the raw concentration values to avoid < 0 post-transformed values.

Telomere length was quantified in peripheral blood mononuclear cells (PBMCs) at the UCSF Blackburn Laboratory. PBMCs were isolated from whole blood by Ficoll Hypaque density gradient centrifugation within 6 hours of blood drawing, cryopreserved in liquid nitrogen, and stored at −80 °C until assay. Genomic DNA was purified in batches using the QIAamp® DNA Mini Kit (QIAGEN, Hilden, Germany, Cat. #51,104). DNA quality criteria were OD260/OD280 between 1.7 and 2.0 and concentration > 10 ng/µL. All samples passed DNA quality check. DNA was stored at −80 °C. The telomere length assay was adapted from the original published method by Cawthon [70, 71], and assay details specific to this study are described in Supplemental Materials. Data are reported as the ratio of the amount of telomere amplification product (T) to that of a single-copy gene (S). This T/S ratio may be converted to base pair units using the formula: bp = 3274 + 2413 × (T/S). The T/S ratio for each sample was measured twice. If the duplicate T/S value and the initial value varied by more than 7% the sample was run the third time and the two closest values were reported. The average coefficient of variation (CV) was 2.1% (± 1.5%).

### Data Analyses

To test our first hypothesis that higher psychological resources would be associated with better biological health, we first performed individual linear regression models for each well-being measure and each biomarker outcome. Values that were three standard deviations above the mean were winsorized to the value of three standard deviations to mitigate potential bias (< 2% of data points). We controlled for BMI and age given the influence of these factors on these biomarkers and following recommendations and previous research [72]; other factors that may influence basal levels of these biomarkers were not relevant for our sample (i.e., we did not have to control for gender as our sample was all women who were non-smokers with limited diversity of socio-economic status). To test our second hypothesis that caregivers would report lower psychological resources and worse biological profiles, we compared the two groups on measures of resources using *t*-tests and compared their biomarker levels by running contrasts on the adjusted means after running regression models that controlled for BMI and age. To test our third hypothesis that the relationship between psychological resources and biomarkers of aging would be different in the context of caregiving, we ran regression models that included an interaction term of the resource measure by caregiver status, as well as by parental stress. We report the change in *R*^2^ from a model with covariates only (BMI and age) to a model with the predictor of interest included to identify the percent of variance explained by each predictor. To compare whether any individual psychological resource is more closely tied to health outcomes than others, we included all resource measures in a single model (for each outcome).

## Results

### Sample Demographics for Women Who Completed the Relevant Study Time Point

One hundred forty-seven women completed the 18-month study visit. Fewer caregivers (*n* = 67) compared to controls (*n* = 80) completed this visit, though there were no differences between women who completed this visit and women who did not on baseline perceived stress scores or demographic factors. Women were, on average, 44 years old, non-Hispanic White (78%), completed bachelor’s degree (87%), married (86%), and had a household income at or above $125,000 (79%). The sample biomarker descriptive statistics are presented in Supplemental Table S1.

### Group Differences in Psychological and Biological Measures

By design, caregivers reported significantly higher levels of perceived stress at baseline (mean = 21.89, SD = 4.66, range = 12–33) than the control group (mean = 15.72, SD = 4.37, range = 7–29), *t*(179) = −9.18, *p* < 0.001, and these group differences remained at the 18-month visit (caregiver mean = 20.55, SD = 5.11, range = 5–30; control group mean = 15.97, SD = 4.88, range = 7–29), *t*(141) = −5.45, *p* < 0.001. Caregivers also reported significantly higher levels of parental stress (caregiver mean = 46.312, SD = 9.43, range = 24–68; control group mean = 36.51, SD = 8.09, range = 19–61), *t*(140) = −6.65, *p* < 0.001. The overall sample mean for parenting stress was 40.85 (SD = 9.95), with 76% of caregivers scoring above this mean, and only 30% of controls. Caregivers reported significantly lower levels on all psychological resource measures compared to controls. Caregivers also had significantly higher levels of HOMA-IR compared to the control group, after adjusting age and BMI, *b* = 0.671, *p* = 0.001. The two groups did not differ on other biomarkers. Group difference results are presented in Table 1.

**Table 1:** Group differences in psychological resources, subjective stress, and biomarkers

Measure	Caregivers	Control	Group difference
	Mean (SD)	Mean (SD)	*p* value
Psychological resource measures			
Eudaimonic well-being (MHC-SF)	33.57 (8.77)	38.84 (8.05)	**< 0.001**
Autonomy	4.0 (0.61)	4.2 (0.52)	0.055
Purpose in life	4.3 (0.64)	4.9 (0.59)	**< 0.001**
Self-acceptance	4.0 (0.79)	4.8 (0.77)	**< 0.001**
Mastery	2.8 (0.55)	3.4 (0.50)	**< 0.001**
Stress measures			
Perceived stress	20.5 (5.1)	16 (4.9)	**< 0.001**
Parenting stress	46.3 (9.4)	36.5 (8.1)	**< 0.001**
Biomarkers			
HOMA-IR^a^	3.36 (0.14)	2.69 (0.13)	**0.001**
CRP^a, b^	1.95 (0.49)	2.52 (0.45)	0.402
IL-6^a, b^	2.26 (0.47)	1.69 (0.43)	0.373
Telomere length (PBMC)^a^	1.16 (0.02)	1.21 (0.02)	0.131

^a^These are predicted means from regression analyses controlling for BMI and age

^b^Untransformed values are presented here for ease of interpretation though in the regression analyses presented in the paper the data was normalized using log transformations prior to analysis

### Psychological Resources and Biomarkers of Aging

Associations between psychological resources and biomarker outcomes are presented in Table 2, and correlations between all main study variables are presented in Supplemental Table S2. The *R*^2^ change between the model with only BMI and age, and the models that include the predictor of interest are included in Table 2. For HOMA-IR, the base model with only BMI and age included as predictors was statistically significant, F(2, 141) = 61.18, *p* < 0.001, *R*^2^ = 0.46, suggesting that this base model accounts for 46% of the variance in HOMA-IR. Each individual resource measure was a significant predictor, except autonomy, adding between 1 and 10% additional variance in HOMA-IR explained. In a model the included all the psychological resource measures along with BMI and age, 59% of the variance in HOMA-IR was accounted for, F(7,115) = 23.75, *p < *0.001, *R*^2^ = 0.59.

**Table 2:** Adjusted regression models examining associations between psychological resources and biomarkers

	HOMA-IR				CRP (ln)				IL-6 (ln)				PBMC telomere length			
	B	SE	*p*	*R*^2^∆^a^	B	SE	*p*	*R*^2^∆	B	SE	*p*	*R*^2^∆	B	SE	*p*	*R*^2^∆
Eudaimonic well-being	−0.235	0.10	**0.018**	0.03	−0.020	0.05	0.690	0.02	−0.005	0.01	0.460	0.01	0.014	0.02	0.404	0.00
Autonomy	−0.068	0.10	0.506	0.01	0.042	0.05	0.402	0.03	0.008	0.03	0.795	0.00	0.024	0.02	0.147	0.01
Purpose in life	−0.295	0.10	**0.003**	0.04	−0.001	0.05	0.988	0.02	−0.017	0.03	0.569	00.01	−0.006	0.02	0.722	0.00
Self-acceptance	−0.313	0.10	**0.001**	0.05	0.012	0.05	0.806	0.02	−0.015	0.03	0.552	0.11	0.015	0.02	0.373	0.00
Mastery	−0.322	0.10	**0.001**	0.10	0.006	0.05	0.909	0.00	−0.016	0.03	0.611	0.01	0.007	0.02	0.662	0.00

Confounds included in these models were age and BMI. Bolded numbers indicate a significant p value of <.05

^a^This number represents the *R*^2^ change; the percent of variance explained by the model with the primary predictor of interest in it minus the percent of variance explained by the model with only BMI and age as predictors

For CRP, the base model with only BMI and age included as predictors was statistically significant, F(2, 140) = 33.65, *p* < 0.001, *R*^2^ = 0.32. When the resource measures were added to the model, there were no significant predictors of CRP. For IL-6, the base model with only BMI and age included as predictors was statistically significant, F(2, 140) = 14.61, *p* < 0.001, *R*^2^ = 0.17. When the resource measures were added, none were significant predictors of IL-6. For telomere length, the base model with only BMI and age included as predictors was statistically significant, F(2, 142) = 5.69, *p* = 0.004, *R*^2^ = 0.07. When the resource measures were added, none were significant predictors of telomere length.

As subjective levels of stress have previously been linked to these biomarkers, and the two groups differed on both measures of subjective stress, we also examined the associations between the Perceived Stress Scale and Parenting Stress Scale and each biomarker outcome with age and BMI as covariates. For HOMA-IR, the Perceived Stress Scale was a statistically significant predictor, F(3, 137) = 44.82, *p* = 0.002, *R*^2^ = 0.49 for the overall model with *b* = 0.055, *p* = 0.002, as was the Parenting Stress Scale, F(3, 136) = 14.61, *p* < 0.001, *R*^2^ = 0.17 for the overall model with *b* = 1.33, *p* = 0.001. Neither measure of subjective stress was associated with CRP, IL-6, or telomere length.

### Psychological Resources and Health in Stressful Contexts

**Fig. 1:**
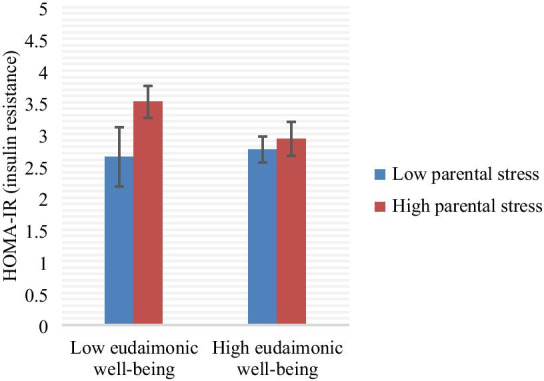
Interaction of eudaimonic well-being (as measured with the MHC-SF) and parenting stress on HOMA-IR

Caregiver status did not moderate the association between any of the resource measures and any of the biomarker outcomes. However, given that not all caregivers report high levels of perceived stress from their parenting role, we also examined whether levels of perceived parental stress moderated the association between psychological resources and biomarker outcomes, regardless of caregiver status (and also controlling for BMI and age). Indeed, we found a significant interaction between eudaimonic well-being (MHC-SF) and parental stress in predicting HOMA-IR, *F*(6, 130) = 27.69, *p* < 0.001, *R*^2^ = 0.56 for the full model, with *b* = −0.019, *p* = 0.036 for the interaction; the change in *R*^2^ between the model without the interaction term and with the interaction term was 0.015. Thus, parental stress was a significant moderator of the relationship between eudaimonic well-being and HOMA-IR. The simple slope for participants’ two standard deviations above the mean of parental stress was −0.046 (*SE* = 0.02, *p* = 0.038), the simple slope for participants at the mean was −0.005 (*SE* = 0.012, *p* = 0.67). The simple slope for participants’ two standard deviations below the mean of parental stress was 0.038 (*SE* = 0.024, *p* = 0.038). To demonstrate this relationship visually, participants were grouped based on whether they were above or below the mean on both eudaimonia and parenting stress; as Fig. 1 shows, women reporting low eudaimonic well-being, high parental stress was associated with greater insulin resistance (n=139; bars in figure indicate standard errors). For women reporting higher eudaimonic well-being, level of parental stress was not associated with insulin resistance levels. This suggests that women reporting high eudaimonic well-being were protected from the negative effects of high perceived parental stress on metabolic health.

**Fig. 2:**
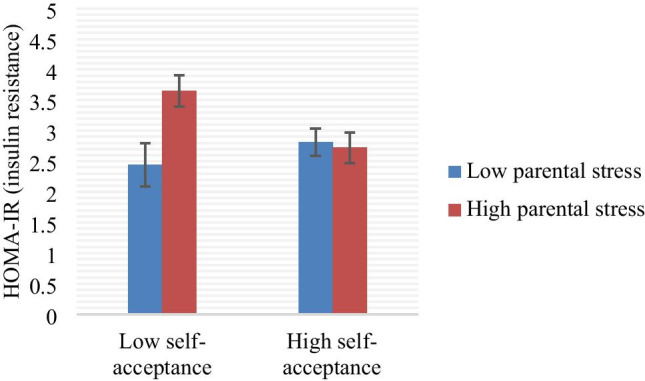
Interaction of self-acceptance and parenting stress on HOMA-IR

We also found a significant interaction of the self-acceptance subscale and parental stress when predicting HOMA-IR, F(6, 130) = 28.32, *p* < 0.001, *R*^2^ = 0.57 for the full model, with *b* = −0.017, *p* = 0.017 for the interaction (controlling for BMI, age, and caregiver group status); the *R*^2^ between the model without the interaction term and with the interaction term was 0.019. The simple slope for the participants’ two standard deviations above the mean of parental stress was −0.514 (*SE* = 0.209, *p* = 0.015), the simple slope for participants at the mean was −0.055 (*SE* = 0.117, *p* = 0.638). The simple slope for participants’ two standard deviations below the mean of parental stress was 0.403 (*SE* = 0.237, *p* = 0.091). This interaction similarly revealed that women with higher parental stress evidenced higher insulin resistance but only if they reported lower self-acceptance. Specifically, as demonstrated visually in Fig. 2, among women reporting low self-acceptance, high parental stress was associated with greater insulin resistance, whereas for women reporting higher self-acceptance, level of parental stress was associated with insulin resistance levels. For visual purposes, a bar graph is used to plot levels of HOMA-IR after categorizing individuals based on whether they reported above the mean or below the mean levels of parental stress, and above the mean or below the mean on the self-acceptance (n=139; bars indicate standard errors). This suggests that women reporting high self-acceptance were protected from the negative effects of high perceived parental stress on metabolic health. There were no other significant interactions between the other resource measures and parenting stress, or between the resource measures and global perceived stress.

## Discussion

Can psychological resources protect women from the wear and tear of chronic parenting stress? We examined the association between five different psychological resources (eudaimonic well-being, autonomy, purpose in life, self-acceptance, and mastery) and indices of biological health in mid-life women. Our sample was specifically recruited to examine differences in these relationships for those experiencing high verus low levels of current perceived stress. Roughly half of the women recruited were high stress maternal caregivers of children with an autism spectrum disorder, and the other half were age-matched mothers of neurotypical children. Compared to the control group, the caregivers reported higher global perceived stress and parenting stress, lower levels of eudaimonic well-being, purpose in life, self-acceptance, and mastery, and had higher insulin resistance. Across the sample as a whole, higher eudaimonic well-being, purpose in life, self-acceptance, and mastery were associated with better metabolic health as indexed by lower insulin resistance. Resources were especially important for those with greater stress. Moderation analyses showed that insulin resistance was worse among mothers with high parenting stress and with low eudaimonic well-being, a pattern also evident for self-acceptance. Framed another way, for women feeling high levels of parenting stress, if they also have high psychological resources, their health was protected. Parenting stress has increased recently due to the COVID-19 pandemic and schooling at home [39]; our findings may have even more public health significance during times when stress-buffering social systems (e.g., time with friends) are reduced.

Our finding of psychological resources and better metabolic control align with previous research that has found a positive association between similar resource constructs and metabolic health in older women [19], including in large national cohort studies [2, 19, 73]. Our work extends these findings to young healthy women. When examining the percent of variance in insulin sensitivity explained by our base model that included the known determinants of age and BMI as predictors, we found that 46% of variance was explained. When adding in each type of psychological resource measured in this study in order to assess effect size, we found that each resource explained an additional 1 to 10% of unique variance. Together, resources explained an additional 13% of variance on top of the variance explained by age and BMI, suggesting that these measures are meaningful additions to predictive models of HOMA-IR.

One pathway explaining the association between both stress and psychological resources on metabolic health may be because both high perceived stress and lack of resources are associated with dysregulated eating behaviors, potentially leading to problems with glucose regulation [74, 75]. Past research has linked stress eating to worse glyciemic control among non-diabetic adults in both an experimental study [76] and utilizing a large cohort study [77]. The role of insulin sensivity and insulin resistance in the association between mood, stress, and disease pathogenesis is a promsing area for future research, with compelling theoretical arguments and burgeoning empirical evidence for the links, as described by Rasgon and McEwen [78].

Counter to our hypotheses, there were no associations between psychosocial resources and systemic inflammation or telomere length. This is in contrast to previous research showing that greater resources are associated with lower systemic inflammation [14–16]. We were also surprised to find no group differences in inflammation or cellular aging indicators between chronically stressed and low stress women, or between subjective stress measures and these biomarkers. The lack of main effects of psychological resources and caregiver status and subjective stress on systemic inflammation and telomere length were surprising given past research. Our null results may be because the women in our sample were relatively young (age 20–50) and healthy. They reported high levels of strong stress buffers, including being married, college educated, and high income. Interestingly, early childhood adversity was linked to shorter telomere length and greater telomere length attrition in this sample, as reported elsewhere [79]. This stress-related finding suggests that while their current adulthood experiences of perceived stress may not be strong enough to create measureable biological change, these women were not immune to stress-related influences.

The caregiver versus control group difference in HOMA-IR suggests that metabolic health may be the first biological system impacted by sustained perceived stress in adulthood. Insulin sensitivity may be a measure that is more responsive to contextual factors before chronic disease sets in for young, healthy, well-resourced women, while systemic inflammation and telomere length may be less influenced. Future research studies with similar samples should consider that HOMA-IR may be a particularly sensitive outcome of stress in healthy mid-life women, especially given previous work linking it to stress eating and showing stress-associated changes as early as childhood.

While there was no significant interaction of eudaimonic well-being as measured by the MHC-SF and caregiver status as we had predicted, we found that there was an interaction of eudaimonic well-being and parental stress. Specifically, eudaimonia was associated with higher insulin resistance *only* among women reporting higher parental stress. Perceived parenting stress, because it is a continuous measure and because there is a range of stress levels in each group, is likely a more sensitive measure of stress than caregiving group status. Although there are no established clinical cut-offs for HOMA-IR, levels above 3 have been suggested to be clinically high insulin resistance—and the women in our study who reported high parental stress and low eudaimonia had mean levels of 3.5, suggesting health risk. Parallel results were found for self-acceptance. Among women reporting lower parental stress, insulin resistance did not differ by level of eudaimonia or self-acceptance. Thus, higher eudaimonic well-being and self-acceptance may be protective factors against higher parental stress.

The specific psychosocial resource that offers the most benefit may depend on the factors of the stressful context. For the specific sample studied here—mid-life women parenting young children and living in a metropolitian area with relatively high resources—self-acceptence may be one particularly beneficial resource. Self-acceptance is explained by Ryff (1989) as holding positive attitudes toward onself and is described as central to positive psychological functioning and self-actualization. In the context of chronic stress, self-aceptance may be beneficial as greater acceptance of all parts of oneself can include accentance of one’s negative thoughts and feelings related to the stressor, in this case, parenting stress. For parents of children with autism, self acceptance may be a buffer to the higher levels of childcare challenges such as a child’s emotion dysregulation. Acceptance of one’s own emotions is an effective strategy for emotion regulation and is the basis of the evidence-based psychological treatment Acceptance and Commitment Therapy [80, 81].

In addition to acceptance of self, acceptance of one’s life circumstances may also be a central resource in the context of chronic stress. In our previous daily-level analyses with this sample, we found that actively engaging in the present moment and accepting the reality of the current moment (versus fighting or rejecting it) is associated with better daily mood, lower stressor-related rumination, and higher daily social connection with one’s spouse [82, 83]. This aligns with past research, psychological theories, and perspectives from wisdom traditions that emphasize the importance of acceptance of one’s current life circumstances for strong emotional health and for self actualization [84, 85]. Having high levels of acceptance means that the constant process of evaluating life and self in terms of “good” and “bad” experiences, thoughts, or behaviors, and then working to avoid the “bad,” is attenuated. Instead, acceptance of how things are allows one to aquire “more flexible and less defensive styles of dealing with difficult thoughts, feelings, or sensations” [85, p. 880]. This approach may allow for an individual to be better able to cope with the uncertainty and greater daily stress that comes with chronically stressful circumstances, ultimately leading to an emotionally stable and healthy way of existing in the world.

There are several limitations of this study. First, despite the longitudinal design of the parent study, the psychological resource measures were not included until the third timepoint, which limited our ability to conduct longutidinal analyses that would allow us to test for causal inference. We also did not include all components of Ryff’s well-being model and thus are not able to make conclusions about which aspects may be the most helpful in buffering the chronic stress of mothering a child with an autism spectrum diagnosis, a key place for future research attention, given the importance of identifying where to target interventions. The study design also did not allow us to examine other aspects of metabolic health such as HbA1c or metabolic syndrome, which have been associated with both subjective stress and eudaimonic well-being. Our results however, suggest that including a broad array of metabolic functioning indices may be a fruitful effort. Furthermore, because the primary predictors were positive psychological factors, stronger associations may be found with indicators of positive biological functioning, such as anti-inflammatory cytokine production or heightened parasympathetic nervous system dominance, another potential area for future research. Our sample is limited in that we are studying a specific form of chronic stress—maternal parenting stress measured at one time point—and different psychological resources may be more or less protective in different contexts and stressors. Future studies may also consider a more specific examination of the other forms of parenting-related stress that participants may be experiencing. The women in our control group may have been caring for a child who struggled in ways other than neurological development, such as being bullied or struggling to to keep up academically for example, which may place a high burden on the parents and explain the lack of group differences on stress-related biomarkers. Another design limitation is that we did not have a measure of social support we were able to use in these analyses, an important limitation given the key role social support plays in stress resilience and its strong relationship to physical health. Finally, our sample was relatively homogenous demographically, with the majority non-Hispanic white, married, well-educated, young, and in a high income bracket, limiting the implications of our findings to other groups, especially those with fewer resources. Finally, these results can also only be generalized to mid-life women; future work should explore how psychological constructs influence metabolic health at different points in the lifecourse.

A major strength of the study is the recruitment of chronically stressed and low stress women using both an objective measure (children’s diagnosis with ASD or developmentally typical child) and subjective measures of stress (global perceived stress and perceived parenting stress). This allowed for an investigation of the role of psychological resources in circumstances for which it is hypothesized that positive psychological constructs would be helpful in remaining resilient to difficult life circumstances. Additional strengths of the study include the size of the sample and the inclusion of telomere length which has received limited attention in the psychological resource literature.

In sum, our results showed that for healthy mid-life women, greater psychological resources are associated with better metabolic health, and that in particular, self-acceptance and eudaimonia are protective in the context of chronic parenting stress. Metabolic dysregulation in the form of insulin resistance may be particularly impacted. Since the relationship between psychological resources and other biomarkers of aging were null, the study results suggest that chronic parenting stress may first impact metabolic health before other indices of aging. Understanding the role psychological resources play in the biological intermediaries of disease development, and which resources are useful under which contexts, will help identify psychological targets for intervention. This study suggests that for chronically stressed mothers, increasing self-acceptance and eudaimonic well-being may be impactful intervention targets.

## Supplementary Information

Below is the link to the electronic supplementary material.

Supplementary file1 (DOCX 13 KB)
