# Supplementary material for: Psychological Resources and Biomarkers of Health in the Context of Chronic Parenting Stress
Source: Int J Behav Med. 2021 Aug 6;29(2):175–87. doi: 10.1007/s12529-021-10007-z (PMC8343363; doi:10.1007/s12529-021-10007-z)
Supplement: Supplementary file 1 — Supplementary file1 (DOCX 13 KB) [file 12529_2021_10007_MOESM1_ESM.docx]

Supplemental Materials

The first subset of participants’ blood from the 18 month blood draw was assayed in 2013 (n=40). These were the first participants who enrolled in the study and due to the multi-year recruitment effort, these participants had completed their 18 month blood draw by the time all other participants had completed the baseline assessment. Thus, this subsample had their 18 month blood draw assayed along with all other participants’ baseline blood draw. The remaining participants had their 18 month samples assayed in 2015, when all participants had completed their 18 month assessment. There was a change in the batch of reference standard genomic DNA used in the qPCR assay from 2013 to 2015, and there were different batches of assay reagents including dNTPs and Taq polymerase. Because of these technical changes, there was a significant difference in the range of values from the sample assayed in 2013 and the sample assayed in 2015. After extensive discussion with the Elizabeth Blackburn Lab scientists (lead: Dr. Jue Lin, Director of Telomere Core, Department of Biochemistry and Biophysics, University of California San Francisco), the decision was made to re-assay 86 samples from one of the 2013 source DNA plates. This plate was randomly selected. Based on those values, a correction factor was then applied to the 2015 data to account for assay drift that had occurred due to the differing techniques at the two assay time points. The correction was a simple linear regression (y=2015 measurement, x=2013 measurement) applied to new 2015 data: 2015 adjusted value = (2015 lab value - .04472)/.8676. This adjusted value data for the 18 month time point is what is used in the analyses reported in this paper. This was only addressed for telomere length, not other biomarkers, because of the procedure used for this assay changed between years.
